# Managing congestion at visitor hotspots using park-level use level data: Case study of a Chinese World Heritage Site

**DOI:** 10.1371/journal.pone.0215266

**Published:** 2019-07-26

**Authors:** Jin-Hui Guo, Tian Guo, Kai-Miao Lin, Dan-Dan Lin, Yu-Fai Leung, Qiu-Hua Chen

**Affiliations:** 1 College of Tourism, Wuyi University, Wuyishan City, Fujian, China; 2 College of Management, Fujian Agriculture and Forestry University, Fuzhou City, Fujian, China; 3 School of Environment and Sustainability, University of Michigan, Ann Arbor, Michigan, United States of America; 4 Parks, Recreation and Tourism Management, North Carolina State University, Raleigh, North Carolina, United States of America; University at Buffalo - The State University of New York, UNITED STATES

## Abstract

Tourist congestion at hot spots has been a major management concern for UNESCO World Heritage Sites and other iconic protected areas. A growing number of heritage sites employ technologies, such as cameras and electronic ticket-checking systems, to monitor user levels, but data collected by these monitoring technologies are often under-utilized. In this study, we illustrated how to integrate data from hot spots by camera-captured monitoring and entrance counts to manage use levels at a World Heritage Site in Southeastern China. 6,930 photos of a congestion hotspot (scenic outlook on a trail) were collected within the park at a 10-minute interval over 105 days from January to November 2017. The entrance counts were used to predict daily average and maximum use level at the hotspots. Results showed that the average use level at the congestion hotspot did not exceed the use limit mandated by the park administration agency. However, from 9:20 am to 12:00 pm, the use level at hotspots exceeded visitor preferred use level. Visitor use level was significantly higher at the hotspot during a major Chinese “Golden Week”. The daily entrance counts significantly predicted the average and maximum use level at the hotspot. Based on our findings, park managers can achieve the management goals by permitting the corresponding number of visitors passing the entrances. The gap manifested the complexities in visitor capacity management at high-use World Heritage Sites and other protected areas and calls for innovative monitoring and management strategies.

## Introduction

UNESCO World Heritage Sites and similar iconic protected areas attract millions of visitors each year. While with visitation generates economic and social benefits, the popularity invites issues such as overcrowding, congestion, and resources degradation. Particularly, when an excessive number of visitors congregate at “hotspots”, it is to result into intensified environmental impacts, safety hazards, and dissatisfied users [1–3]. Since significant resources, such as magnificent views or iconic geological features, are typically near these hotspots, their overuse and potential spillover effects on the adjacent resource areas are causes of concern. In China, there are 53 World Heritage Sites and more than 5,000 other types of parks and visitor attractions which attract billions of visitors annually, resulting into severe concerns of congestion and safety hazards related to tramping and falling [4–7]. Indeed, avoiding excessive visitors over-staying at these locations simultaneously, here has been a common management challenging many World Heritage Sites, especially in China with such a large population.

The congestion in park settings can be seemed as too many people or vehicles gathering at a crowded place, while crowding highlights whether there are too many people by the personal judgment, which is a negative and subjective evaluation of a use level based on visitor perceptions [8–10]. Different approaches have been used to mitigate crowding and congestion at hotspots, such as educating visitors to avoid peak time, locations and improving infrastructures to increase capacity and managing the use level when use limits were set at park level [11]. In China, all parks and the protected areas are mandated to devise a use limit developed by The National Guideline for Establishing Maximum Carrying Capacity for Parks (NGEMCC) which requires that each visitor should be able to occupy at least 0.5–1 m^2^ on the trail [12–14]. Within this range, park managers may devise their carrying capacity limits based on the parks’ unique physical, social and biological characteristics. The priority of the guideline is to ensure visitor safety and alleviate congestion[14]. Many managers and researchers have noted that regulating visitor access on public lands is a contentious issue, but they didn’t address congestion issues at hotspots in parks based on this guideline[15–17]. As a matter of the fact, to gain public support for a use limits proposal [12], managers need to establish an empirical relationship that predicts how congestion and crowding levels at hotspots are responsive to different use limits applied. However, the lack of empirical evidence to describe the relationship between use level and crowding is a vexing problem for managing visitors [18].

We acknowledge that the number of visitors does not equal with crowding severity. As advancement in visitor use management research, many researchers conceptualize crowding as a visitor subjective evaluation. Adopting this conceptualization helps managers to clarify the goal for managing use levels. While empirical studies have also found little statistical relationships between visitor use level and the experience quality. An explanation is that there exist many other factors, such as individual place attachments, activity types, and personalities, contributing to the perception of crowding[14, 19]. In practice, using proxy evaluative indicators, such as the number of people at one time(PAOT) at the hotspot or persons per viewscape (PPV) on trails, allow managers to quickly evaluate the conditions and respond to potential issues of crowding [15]. With the multi-scale use level data, park managers could establish a link between visitation at park scale and at site scale to guide their long-term and short-term management decisions. Yet, seldom evidence was found to deal with the crowding at site scale by monitoring total use level in Western countries and China. The reason may be the difficulty to get the accurate data or tools to link these types of data[15–17].

Different kinds of technology have been applied to address the park visitor use management. The automated traffic counter was applied to examine the total number of visitors in United Stated Forest Service camp grounds by field personnel [20], it also was used to analyze the number of visitor along the Carriage Roads in Acadia National Park [15]. Infrared technology on multiple-use trails was applied to reflect variability in the proportion of exiting day hikers across time of the day and the trail of use within Grand Canyon National Park [21]. More recently, the real-time and image-processing monitoring system was applied to underground station streams [22], urban communications [23] to estimate visitor flow and density. Nevertheless, they were seldom adopted to address crowding problem in most natural scenic spots, especially in Eastern Asia to date. In China, a large number of high-resolution cameras were installed at natural and cultural parks with high visitor use by park managers in China to manage visitor use and protect the resources at multiple hot spots. High-resolution 24/7 cameras provide opportunities to expand and upgrade the existing visitor monitoring toolkit. Park managers who battled with visitor congestion at multiple hot spots daily could use the technologies to obtain visitor use data for the entire park and at specific sites. But similar research is rare in Eastern Asian countries, such as China with a high population density and large visitation volume at parks and the protected areas.

The objectives of this study are to manage congestion at visitor hotspots using park-level use level data acquired by high-resolution 24/7 cameras at World Heritage sites. Specifically, 1) to identify the empirical relationship between total use level and use level at hotspots in Mt. Wuyi National Park, and 2) to derive recommended use limits based on the identified relationship.

## Methods

### Park management in China

**Fig 1:**
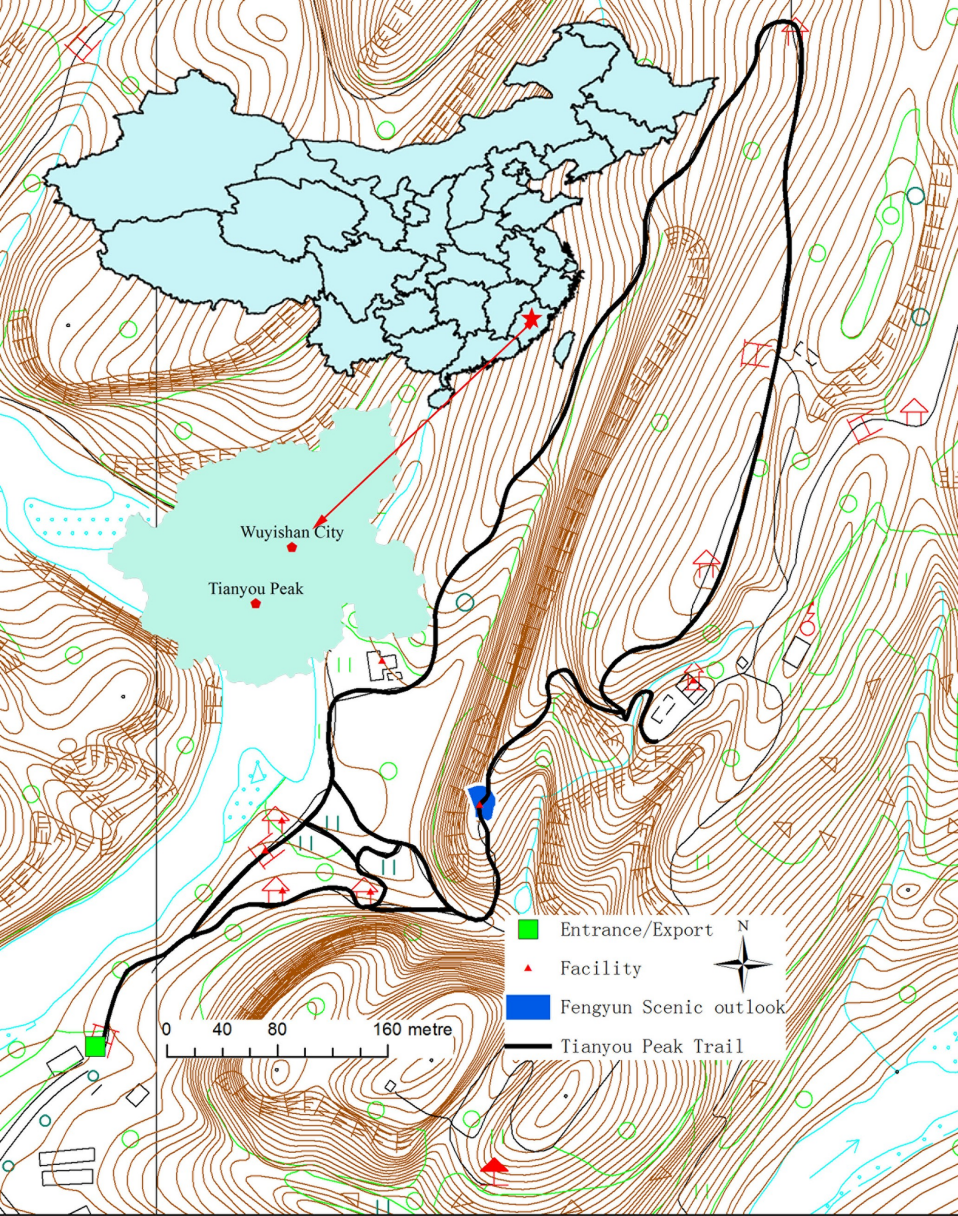
Trail condition and location of Tianyou Peak at Mount Wuyi National Park, China.

Mount Wuyi National Park(27°32′36″~27°55′15″N, 117°24′12″~118°02′50″E) is in southeast of China (Fig 1), approximately covering about 990 km^2^. It was designated as a World Natural and Cultural Heritage Site in 1999 for its outstanding biodiversity value, scenic beauty and cultural significance. Close to major population centers along the East-coast of China, the park faces constant pressure from high use volume and congestion on popular trails.

The park employed a ticket-checking system that monitors the number of visitors entering the park. Managers can impose desired use level limits through controlling how many tickets have been sold. The ticket-checking system at entrances help keep visitation records. High-resolution cameras at specific sites feed real-time images to managers, allowing managers to sharp respond if they observe a safety hazard. Together, the ticket-checking systems and cameras could obtain use-level data for the entire parks and specific sites.

**Fig 2:**
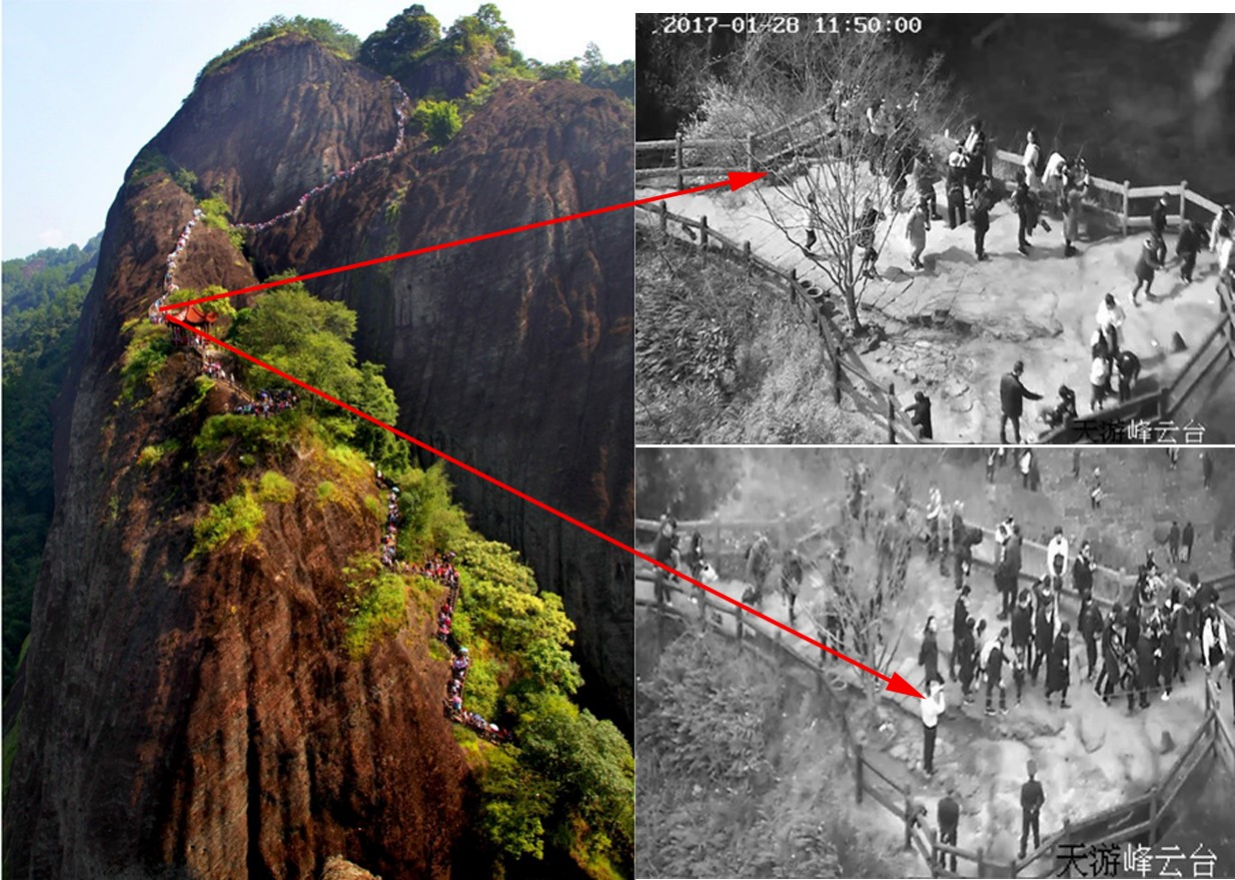
Illustration of photos of Banshan Pavilion on Tianyou Peak Trail in Mount Wuyi National Park.

Tianyou Peak Trail with 1.5-m-wide stone steps ascending 520 meters to the peak is one of the most popular attractions within the park. The outlook Banshan Pavilion rising about 200-meters from the trailhead is the most congested hotspot along the trail (Fig 1 and Fig 2). Many visitors stop at this outlook to rest, enjoy the view and take photos. Thus, visitor congestion occurs often along the trail within the peak time during the holidays according to management experiences reflected by the park managers. Besides, high daily usage on weekends, managers estimated that over 20,000 tourists visited the trail every day during the a-week-long Chinese New Year Holidays and Chinese National Day Holidays. These two important holidays, commonly referred to as the “the Golden Week”, are critical times for generating tourism revenue each year. Concerned with high use volume and congestion along the trail, the park managers decided to install a high-resolution camera on the top of Tianyou Peak to monitor the visitor use climbing along the trail in 2016.

A high-resolution cameras image based real-time monitoring system (Inspur, China) consisted of camera selection, transmission routing design and surveillance center and was presented and implemented in Tianyou Peak for estimating visitor use on the Banshan Pavilion outlook (Fig 2). The resolution of 1.3 million pixels achieved the object tracking the visitor from the picture. The cameras data transmission application was based on TCP/ IP and the GPRS-based data distribution software in accordance with the system. The cameras could rotate or be set manually and take images of multiple sites surrounding the Peak during park’s operation time. The long distance between the camera and the outlook makes it almost impossible to identify individuals in the photos to protect the visitor privacy.

### Data collection

We collected use level data for the park and at the Banshan Pavilion outlook from the park’s camera system from January 28^th^ to November 10^th^ 2017. In order to make data more representative, we worked with the park staff to keep the camera pointing to the outlook for one full day at a time at 3-day intervals, totally10-12 days per month. Consequently, photos were collected for a total of 105 days. The photos were captured by surveillance at 10 min intervals from 7:00 am to 5:00 pm, producing 61 photographs per day to estimate average use level at the outlook throughout a whole day. A total of 6,385 photographs were collected in all 105 days with a few photos missing due to equipment maintenance and occasional malfunctioning (Fig 2). 8 research assistants worked in pairs to count the number of visitors in each photo, with one assistant counting and the checking accuracy. The photos captured 73,158 visitors in total. In addition, entrance counts data were provided through ticket-checking at park entrance for the same days.

The preferred, acceptable and limiting standards were used to express visitors' perception of crowding, and judged the acceptable level of crowding according to crowding norms. The preferred standard refers to resource and/or social conditions that persons would prefer to encounter during their park experience. And the acceptable standard can be defined as the minimum acceptable state of either resource and/or social conditions [2]. While the limiting standard can be defined as the maximum number of visitors which can be accommodated in the parks and other protected areas on the premise of guaranteeing the safety of visitors or resources [14]. The visual-based questionnaires were designed including the acceptability of different levels of crowding. The questionnaire survey was administered for 5 weekends from November 5th to December 18th in 2017 under permission from the Laboratory Ethics Committee of College of Tourism, Wuyi University. 465 Visitors were surveyed randomly on the site when they were visiting the summit of the Peak, which is about 30minutes from the outlook. After the oral consent was informed from the respondents, they were asked to rate the acceptability of photographs showing a range of visitors, marking from the most unacceptable (-4) to the most acceptable (+4) and the neutral point of 0. According to the demographic characteristics of the respondents, female tourists accounted for 50.7% of the total number of tourists, 43.9% of them were 21–35 years old, 40.5% were 36–60 years old, 53.4% were enterprise personnel, 17.6% were government personnel and 10.8% were students.

### Data analysis

We first conducted a descriptive analysis on the use level of the outlook leading to Tianyou Peak. We calculated visitor use level by averaging people per photo and visualized the visitor use level at the outlook over time. We then compared the average visitor use level against three crowding standards, that is, limiting standard, the acceptable standard and the preferred standard. We did a separate analysis on the temporal use level distribution for data collected during the Chinese New Year Holidays from January 28^th^ to February 3^rd^, 2017 and China National Day Holidays from October 1^st^ to October 7^th,^ 2017.

We conducted linear regression statistical analysis in order to link the use level of the entire park with that of the outlook. Since the entrance counts were only available on the daily level, we aggregated the people per photo data to the daily level. Specifically, we summed up people per photo collected in the same day and divided it by the number of photos collected in that day, obtaining the average number of visitors at the outlook at any given time in a day (hereafter *average use level)*. We also obtained the maximum number of visitors at the outlook at a given time in a day (*hereafter maximum use level)*. We fitted one model with the daily entrance counts as independent variable average use level as dependent variable, and another model with the daily entrance counts as independent variable and maximum use level as dependent variable.

## Results

### Temporal visitor use level

Questionnaire survey results showed that park visitors to Tianyou Peak preferred less than 27 people at the outlook (*hereafter preferred standard*), while they considered less than 45 people at the outlook acceptable (*hereafter acceptable standard*). The 14-meter-long and 4-meter-wide Banshan Pavilion covers 56 m^2^ through field observation. We evaluated that each visitor should occupy at least 1 m^2^ at the outlook considering the Situations based on NGEMCC. We calculated that the maximum number of visitors at the outlook at any given moment should not exceed 56 people for the safety (*hereafter limiting standard*).

**Fig 3:**
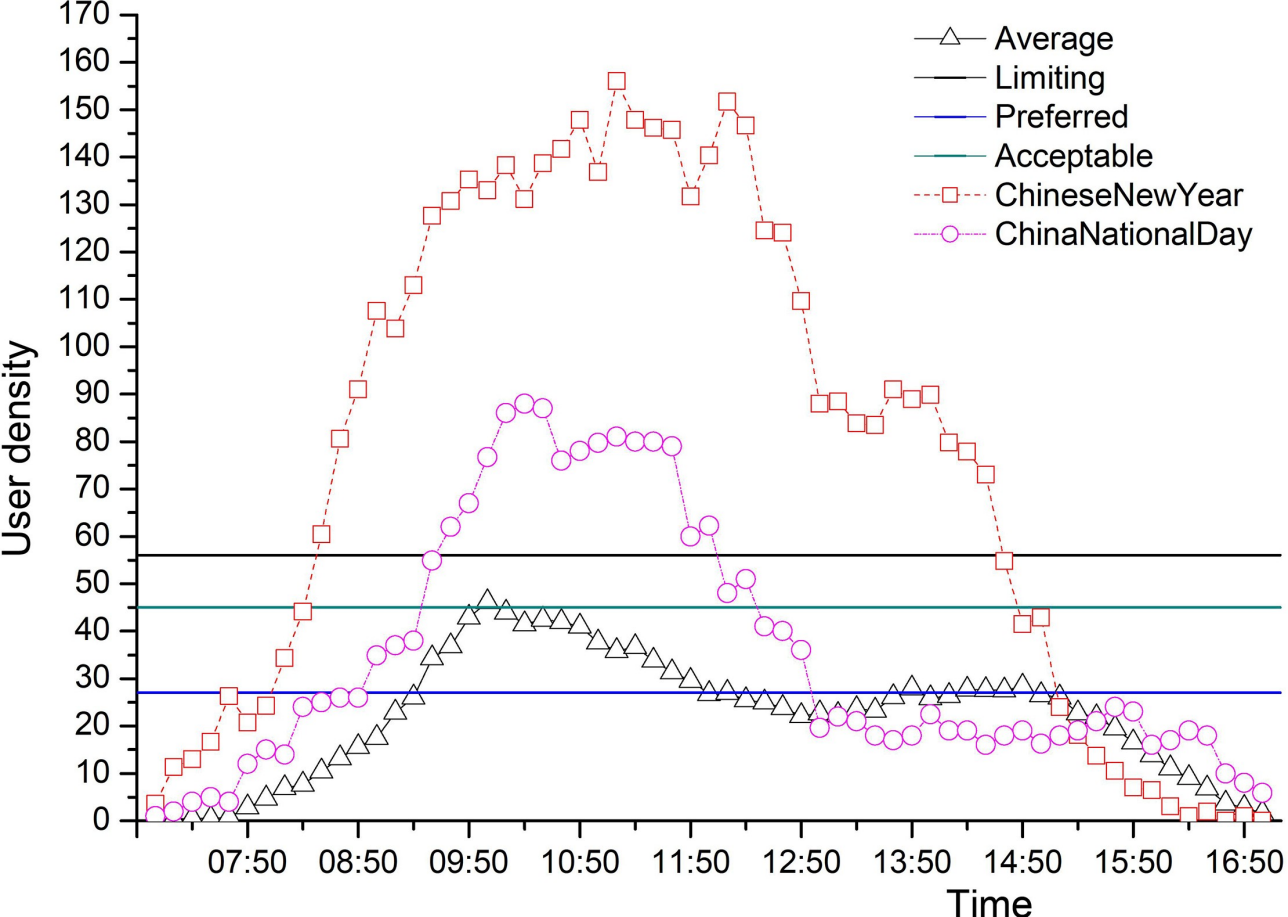
Use level at Banshan Pavilion on the Tianyou Peak Trail at Mt. Wuyi National Park, China. Average: Daily average visitor use level in normal times, preferred: Visitor preferred standard; Acceptable: Visitor acceptable standard; Limiting: Limiting standard; Chinese New Year: Visitor use level over the Chinese New Year Holidays; China National Day: Visitor use level over the National Day Holidays.

The daily temporal visitor use level at the outlook against three crowding standards are shown at Fig 3. During normal days, the average use level begins to grow at 8:00 am and a peak comes out at 10:00 am, which never exceeds the limiting standard and rarely exceeds the visitor acceptable standard but exceeds the visitor preferred standard. The visitor use level keeps nearly at a constant from 12:00 pm to 15:30 pm and then falls quickly till the end. During this period the average use level never goes beyond the visitor preferred standard. However, during the Chinese New Year Holidays, visitor use level at the outlook peaked at 10:50am which exceeds the limiting standard by 178% and exceeds all three standards nearly from 8:40 am to 14:40 pm. During Chinese National Day, the use level peaked at 11:20am which exceeds the limiting standard by 64% and goes beyond all limiting standards from about 9:20 am to 12:00 pm (Fig 3).

### Correlation between use level for the entire park and at the trail

**Fig 4:**
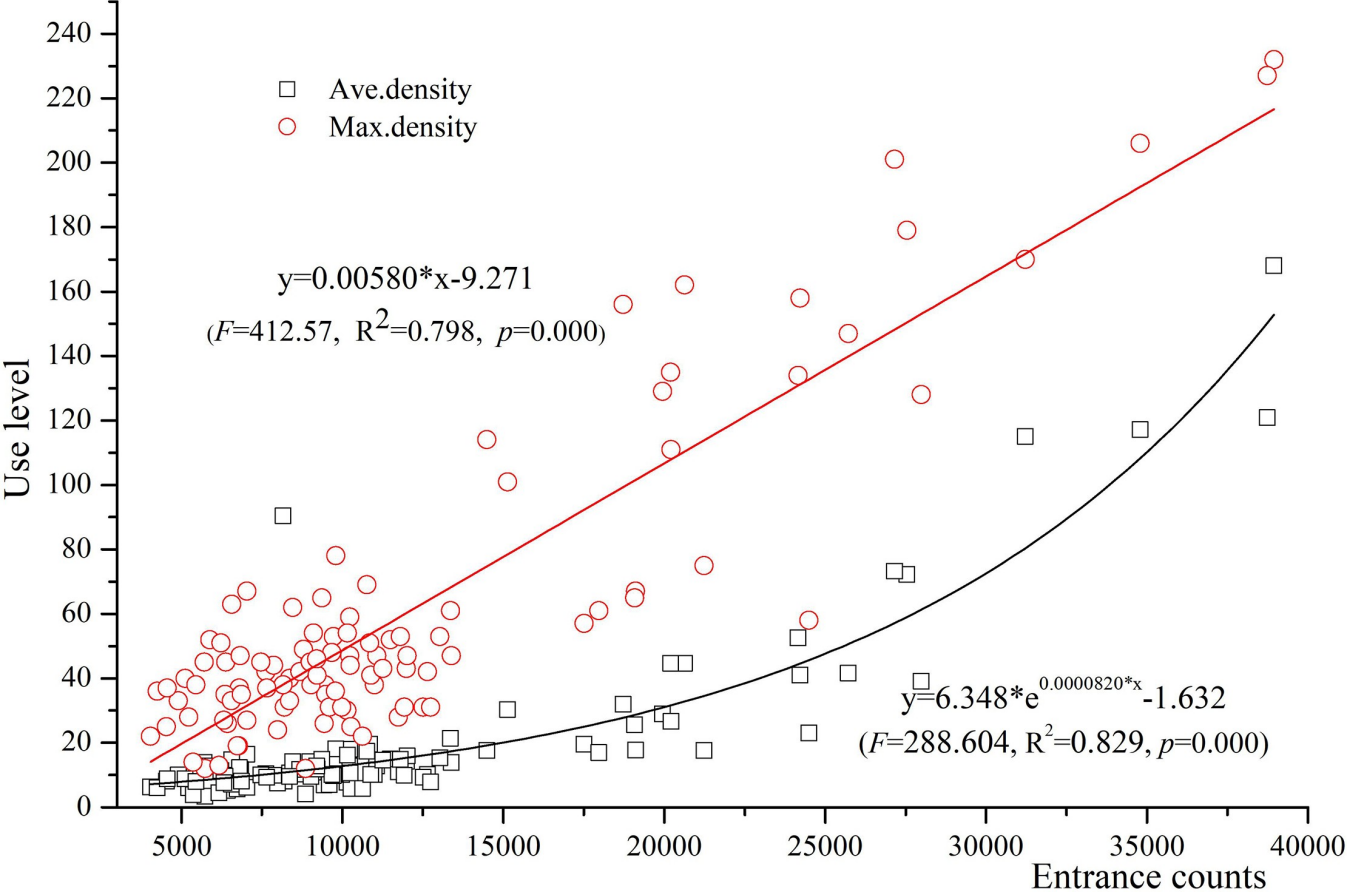
Model results of using entrance counts to predict average use level and maximum use level at the outlook.

The linear regression analysis revealed a significant relationship between the use level for the entire park and at the outlook. The entrance counts significantly predicted daily average visitor use level of the outlook (*F* (1,105) = 288.604, *p-value* < .000, *R*^*2*^ = 0.829). For every additional 1,000 visitors passing through the entrance, on average there will be about 5 more visitors at the outlook at a given time (Fig 4). The entrance counts also significantly predicted the maximum use level at the outlook (*F* (1,105) = 412.57, *p-value* < .000, *R*^*2*^ = 0.798). For every additional 1,000 visitors passing through the entrance, there will be 6 more visitors at the outlook when the uses of the hotspot peaks (Fig 4.).

### Classification of congestion degree

Using the regression model, we calculated at most how many visitors passing by the entrance daily should be permitted so that the average and maximum use level at the outlook will not exceed the crowding standards (Table 1).

**Table 1:** Threshold values of entrance counts for combinations of average use level and maximum use level values at the trail.

Use Level	Preferred Use level	Acceptable Use level	Partially congested	Congested	Very Congested	Extremely Congested
Entrance counts (Z)	Z<6245	6245≤Z <9245	9245≤Z <11078	11079≤Z <19764	19764≤Z <28764	28764<Z
Ave. Use level (X)			X<45	X<45	45≤X<56	56<X
Max. Use level (Y)	Y<27	27≤Y<45	45≤Y<56	56<Y	56<Y	56<Y

1. *Preferred*, when the number of visitors passing by the entrance is less than 6,245, the maximum use level and the average use level at Banshan Pavilion meet the visitor preferred standard. It means congestion never occurs during a whole day.
2. *Acceptable*, when the daily number of visitors passing by the entrance is between 6,245 and 9,245, the maximum use level at the outlook will exceed the visitor preferred standard, but meet the visitor acceptable standard. It means congestion may occurs for a short time but visitors feel acceptable.
3. *Partially Congested*, when the daily number of visitors passing by the entrance is between 9,245 and 11,078, the maximum use level at the outlook will exceed the visitor acceptable standard, but meets the limiting standard. It means congestion only occurs during peak hours but safety management can be guaranteed with any management measures.
4. *Congested*, when the daily number of visitors passing by the entrance is between 11,078 and 19,764, the maximum use level at the outlook will exceed the limiting standard while the average use level meets the visitor acceptable standard. It means congestion takes place during peak time, but on average visitors can accept the occasion.
5. *Very Congested*, when the daily number of visitors passing by the entrance is between 19,764 and 28,764, the maximum use level at the outlook will exceed the limiting standard but the average use level meets the limiting standard. It means extreme congestion occurs, but on average visitors’ safety can be guaranteed if management measures are taken actions.*6)Extremely Congested*, when the daily number of visitors passing by the entrance is over 28, 764, the average and maximum use level at Banshan Pavilion will both exceed the limiting standard. It means whatever actions were taken, congestion cannot be alleviated effectively.

## Discussion

### Temporal visitor use level at the hotspot

Results based on the camera-captured monitoring data showed that the temporal visitor use level between normal times and the two golden week holidays both presented a single peak distribution. The high use level concentrated during 9:00am -12:30pm. An explanation is that visitors preferred to climb the trail to enjoy the Danxia landscape scenery in the early morning. Another reason could be the special landform as the Peak is composed of a great rock without tree shade along the trail. Thus, visitors prefer to climb the trail avoiding exposure under the sunshine.

Meanwhile, results indicated that there is a distinct difference of the temporal visitor use level between normal times and the two golden week holidays. The visitor crowding was very high in two golden week holidays because of the influx of visitors. Prior to this study, park managers could not adopt the proactive management decision making because of lacking the quantitative relationship between the entrance counts and the crowding level at the hotspot. Furthermore, visitor swarming into the park entrance were not well informed about the congestion level at the hotpots for the lack of necessary information. Therefore, the quantitative relationship between the use level of the entire park and that of the hotspot was urgently established to predict the crowding level at the hotspot during peak seasons.

### Links between the use level of the entire park and that of the hotspot

Quantitative information on the hotspot and intensity of visitor use can alert park administrators to manage areas prone to congestion, crowding or safety concerns [19]. Statistical modeling using regression analysis is an effective use of visitor use monitoring data. Results suggested that the relationship between the use level of the entire park and that at the hotspot, once established, can predict congestion level of a tourist hotspot and evaluate compliance or violation of pre-determined use limits based on different management objectives. Only a few researches have tried to predict the visitor use level at the hotspots in western countries [15, 24–26]. The study indicated that establishing links between the entire park and that of the hotspot is feasible in China with large population context. Furthermore, a great majority of parks in China charge fees by checking tickets at the entrances which make it easy to control the total use level to achieve the anticipated management objectives. Therefore, the findings provided Chinese park managers with information to help alleviate congestion, reduce crowding and improve safety conditions at the hotspot under park-scale use limits.

### Visitor capacity management at the hotpot

Visitor capacity research is growing in China [27–30]. However, most of these studies focused on park-scale use level, lacking attention to site-scale use level. The study helped to fill in this gap. This paper compared the visitor use level and the threshold values of entrance count and the results showed that the daily entrance counts significantly predicted the average use level and maximum use level at the hotspot. The use level at the outlook exceeded limiting standard for most of the day during the Chinese New Year Holiday. For most time of an average day, use level at the hotspot did not exceed limiting standard, visitor acceptable standard, or the visitor preferred standard, except for a short period when the use level at the hotspot exceeded the visitor preferred standard. Therefore, less than 6,245 to meet the visitor preferred standard, while the number of visitors permitted each day should be less than 28,764 to keep the visitor safety. If visitor use level exceeded the thresholds, the park management must be faced with the great challenge in countering safety and mitigating impacts of overcrowding.

### Management implications

This study demonstrated how managers could couple camera-captured monitoring data with entrance counts to manage visitor capacity at World Heritage Sites and parks with high use volume. Different congestion scenarios will require different management strategies. For example, in the *Partially Congested* scenario, the maximum number of visitors at the congestion hotspot will exceed visitor acceptable standard, but the average number of visitors at the hotspot will not exceed the acceptable standard. Managers could focus on helping visitors to avoid anticipated peak hours, using behavioral change approaches such as time-based ticket discount for those who visit the park early in the morning. Information may also help visitors avoid hours prone to congestion, such as using a mobile app to distribute real time images of hotspots. In the *Extremely Congested* scenario, park managers should prepare the fast-response protocol.

Cameras could help park managers at other World Heritage Sites to monitor use level at congestion hotspot. Limited park budgets, policies and regulations, or unique size and shape of the congestion hotspots may prevent managers to employ cameras to monitor use level. For example, processing visual data may be labor intensive. We had 8 research assistants counting people per photo for approximately 346 hours. Crowdsourcing such as Amazon Mechanic Turk may be another way to complete the counting task [31]. Violating privacy may be a concern for some cultures. The camera was set up on the top of Tianyou Peak at Mt. Wuyi National Park. The long distance between the camera and the outlook makes it almost impossible to identify individuals in the photos. Managers at other parks who are interested in using cameras for visitor monitoring could carefully design the distance and angle of the camera to avoid invading individual privacy.

### Limitations and future studies

This study contributed to the understanding of visitor capacity at highly populated Asian countries through focusing on visitor monitoring and the number of people at one time at attraction sites. We did not examine other important visitor experience indicators such as the amount of recreational impacts along the trail or maximum waiting time on the trail [32]. We acknowledge that visitor monitoring using camera and entrance counts should complement other types of planning and monitoring strategies in visitor capacity management. We select days to collect camera-captured data with systematic Sampling. There were no reasons to suspect we introduced bias to our data through sampling. The availability of entrance counts limited our regression analysis. We aggregated people per photo to variables at the daily level–the average visitor use level and maximum visitor use level. Future studies could improve the regression model through using a finer time scale, such as linking entrance counts per hour with the number of visitors at any given time at attraction sties. The results supported a correlation between park use level and site use level, but it did not explain the mechanism behind this link. Future research by using GPS and agent-based modeling could help identify visitor movement patterns then predict when congestion occurs on the trail.

## Conclusions

UNESCO World Heritage Sites and other iconic protected areas face the constant tension between visitor use and protecting environment and visitor experiences. Monitoring use level is a core management strategy for visitor carrying capacity. We found camera-captured data are useful to monitor use level at a congestion hotspot within a high-visited World Heritage Site in southeast China. The average and maximum use level at the hotspot could be predicted by entrance counts, suggesting direct relationships between park-scale use level and site-scale use level. Our results suggested park managers should not rely on a single number to manage congestion at hotspots. Instead, park managers need to build a broader decision-making process including multi-scale visitor monitoring. We need more researches to advance our understanding of visitor capacity at different cultural and management contexts and to help World Heritage Sites to address the congestion problem.

## Supporting information

S1 Data Use level at Banshan Pavilion on the Tianyou Peak Trail at Mt. Wuyi National Park, China, showed in Fig 3. (XLS)

S2 Data Model results of using entrance counts to predict average use level and maximum use level at the outlook showed in Fig 4. (XLSX)
